# The Hygiene of Sleep

**Published:** 1900-08

**Authors:** 


### The Hygiene of Sleep.

Eight hours of sleep is necessary for the average individual. The energies of the body do not consist of intangible essences. They are not mysterious forces separate and distinct from matter. A living cell, well stored with energy, shows a great number of minute granules in its interior, and is plump and normal in appearance. An exhausted cell shows the number of granules greatly diminished or absent, and the cell itself has a shriveled appearance. It is in sleep that these energy granules are produced through the assimilation of food taken during the day. During sleep, the amount of oxygen in the body is replenished.

The best sleep is to be secured with a rather low pillow of hair or cotton, or still better an air pillow. The bedclothes should be light. The most healthful plan is for each individual to have his own bed.

The sleeeping room should be thoroughly ventilated, and at a temperature several degrees below that of ordinary working or living room, not higher than 55° or 60°in winter. The best sleep is secured when the stomach is not occupied with digestion. Sound sleep is often disturbed by an overloaded stomach. This condition is also often the occasion of frightful dreams and exhaustive discharges during sleep. Disturbed sleep or insomnia may be relieved by a wet girdle, or by a needle bath before retiring.—*Good Health.*

